# Supplementary material for: Frontoparietal network topology as a neural marker of musical perceptual abilities
Source: Nat Commun. 2024 Sep 17;15:8160. doi: 10.1038/s41467-024-52479-z (PMC11408523; doi:10.1038/s41467-024-52479-z)

Supplementary Material

Frontoparietal network topology as a neural marker of musical perceptual abilities

M. Lumaca*, P.E. Keller, G. Baggio, V. Pando-Naude, C. J. Bajada, M.A. Martinez, J.H. Hansen, A. Ravignani, N. Joe, P. Vuust, K. Vulić, K. Sandberg

Supplementary Figures

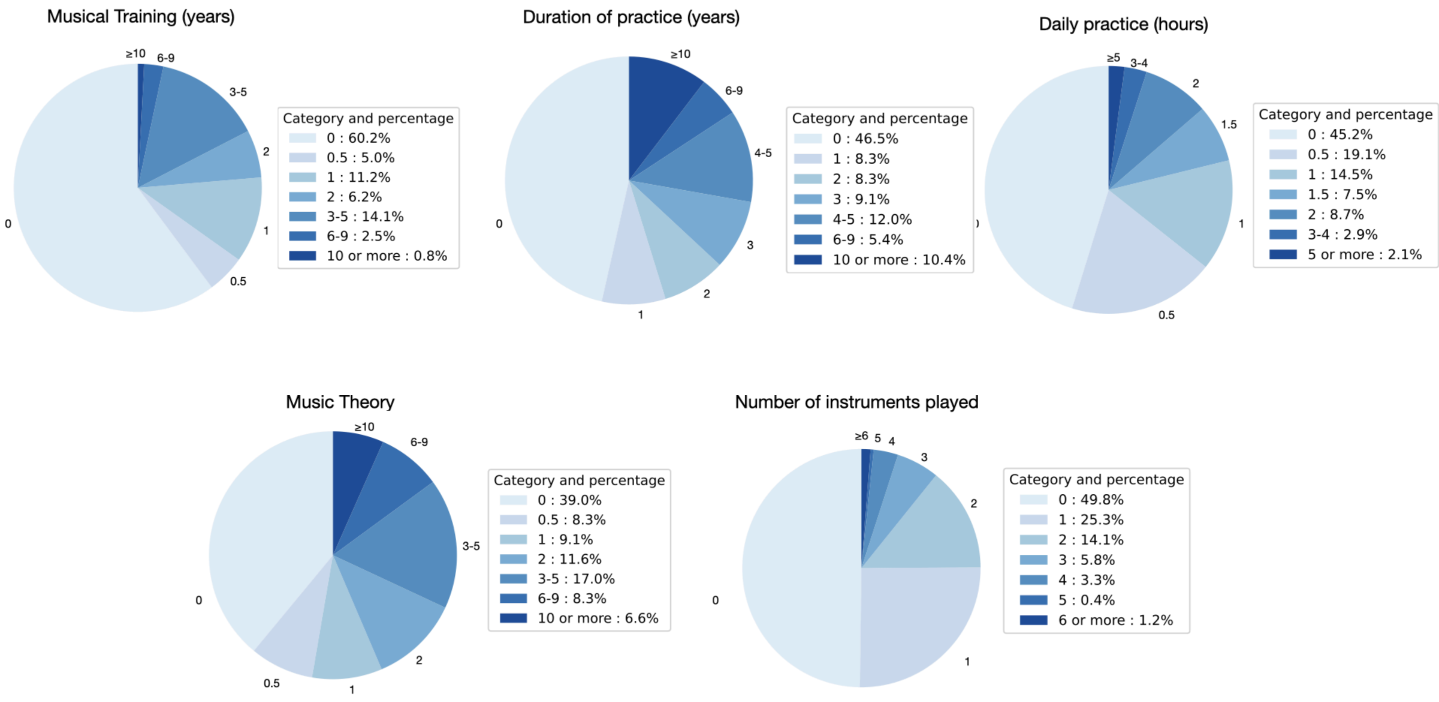

Supplementary Figure 1. Pie charts depicting the distribution of musical training duration and number of instruments played among participants across the Gold-MSI musical training subscales (N=241). Percentages in the legend indicate the proportion of participants categorized by the relative subscale.

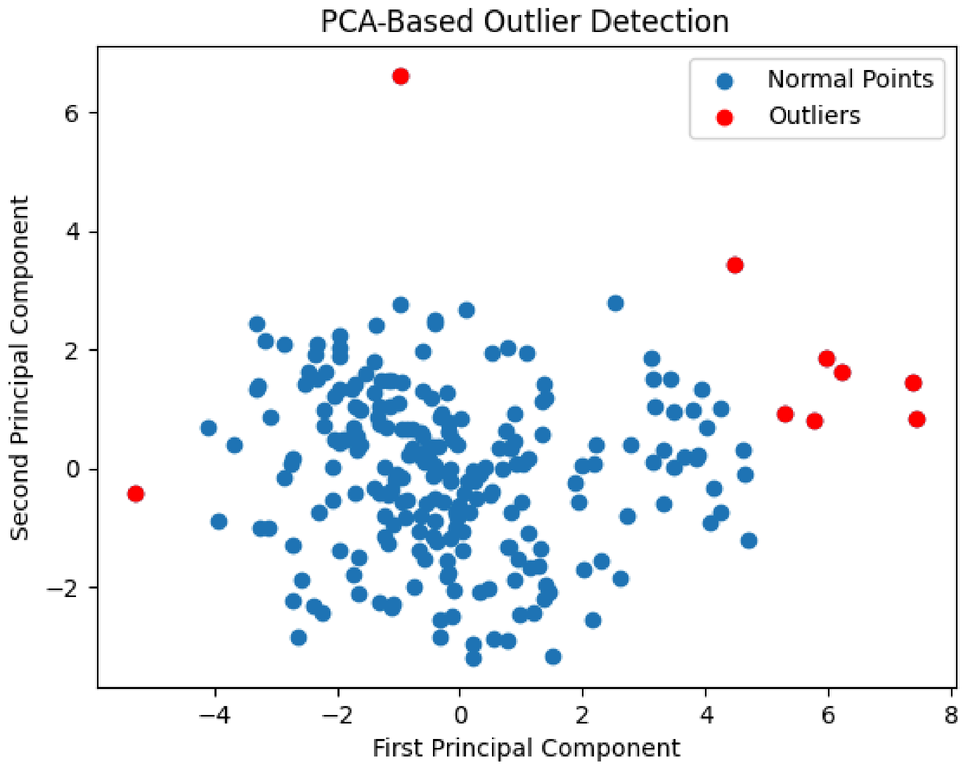

Supplementary Figure 2. Scores from the Musical Ear Test (MET) - encompassing total, rhythm, and melody - and Goldsmiths Musical Sophistication Index (Gold-MSI) subscales were standardized via z-score normalization to ensure equal weighting of variables. The resulting dataset was reoriented to align with principal axes of maximum variance. Outlier detection involved calculating Euclidean distances from the origin in the PCA plane, with primary emphasis on the first two components. Points exceeding two standard deviations from the mean Euclidean distance were designated as outliers. Each point in the scatterplot corresponds to one participant (N=241).

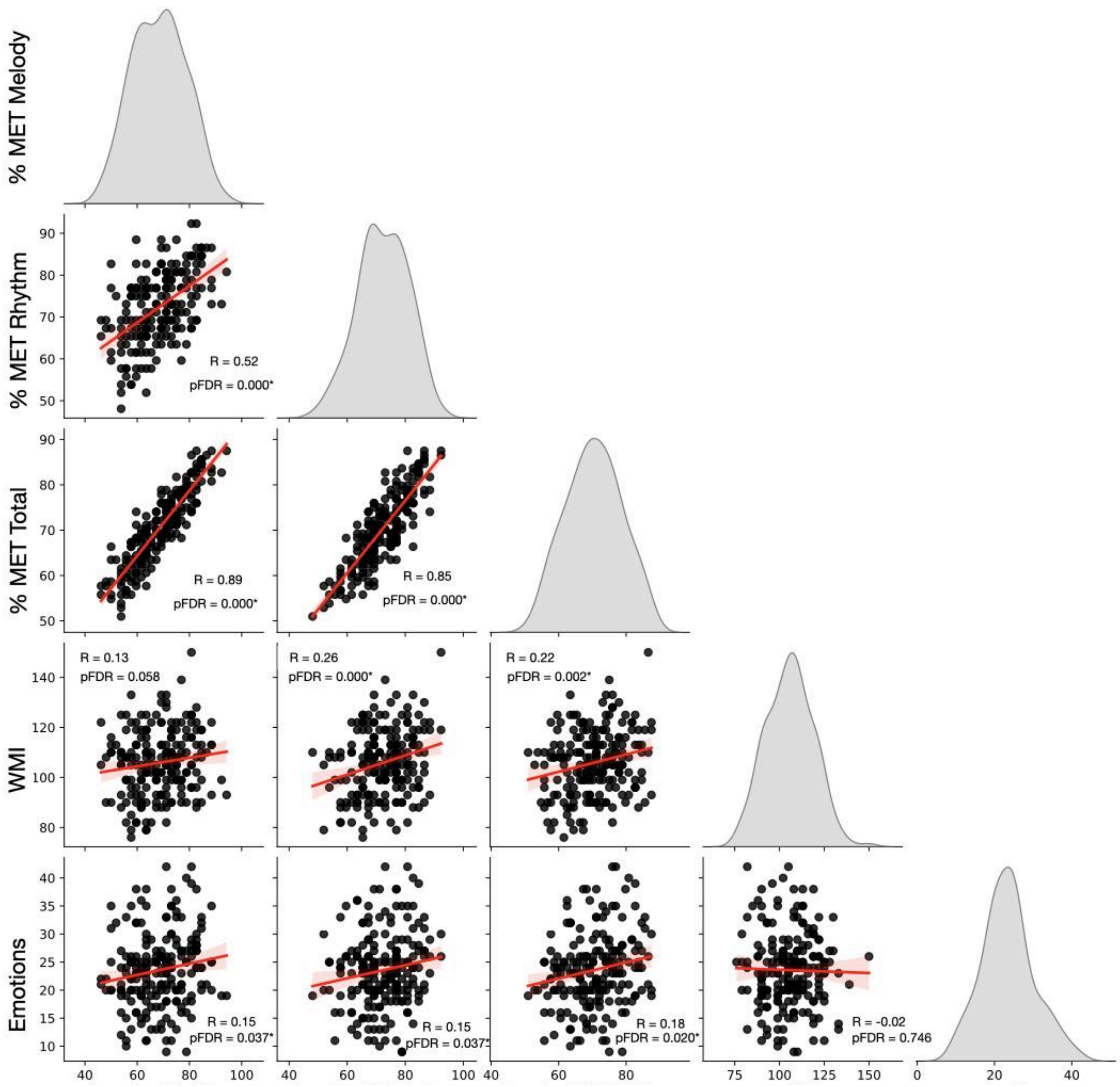

Supplementary Figure 3. Seaborn pairplot displaying Pearson's statistical correlation between behavioural and cognitive scores, as well as self-reported emotional responses to music. Scatterplots illustrate pairwise relationships between variables (percentage of MET melody score, percentage of MET rhythm score, percentage of MET total score, Working Memory Index from WAIS-IV, and Emotions F5 subscale from Gold-MSI) with annotations for Pearson correlation coefficients and adjusted p-values (pFDR). Diagonal kernel density estimates provide univariate distribution views of each

variable. Each plot displays 201 individual data points, corresponding to the number of participants from whom cognitive measurements were also collected.

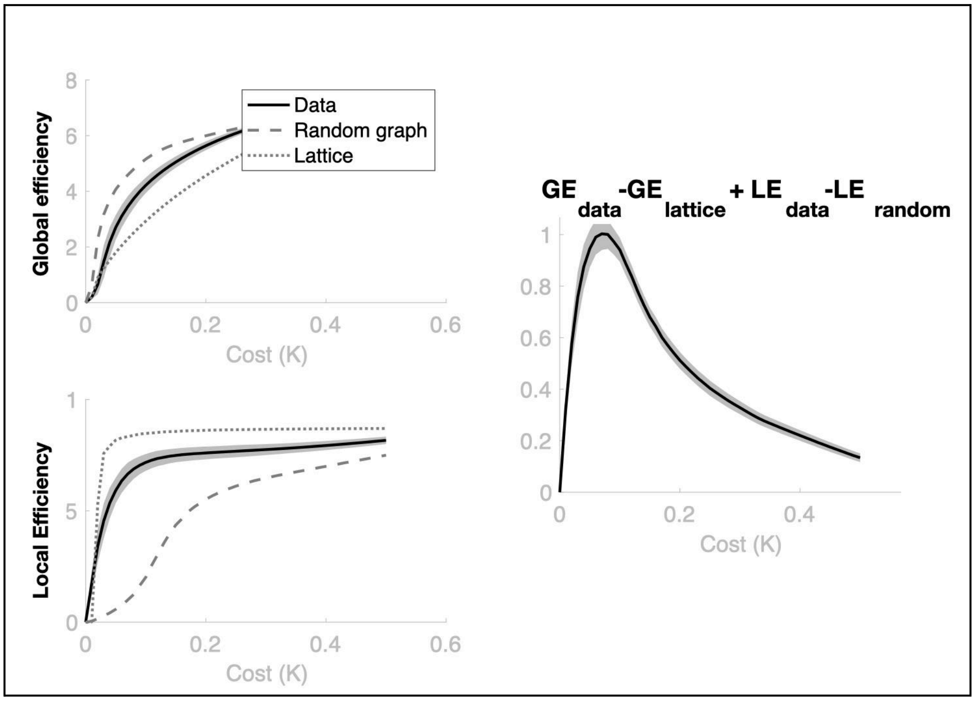

Supplementary Figure 4. Global and local efficiency of the functional network as a function of the cost threshold (k). The network exhibits small-world properties when it shows high global efficiency compared to a lattice graph and high local efficiency compared to a random graph (N=241).

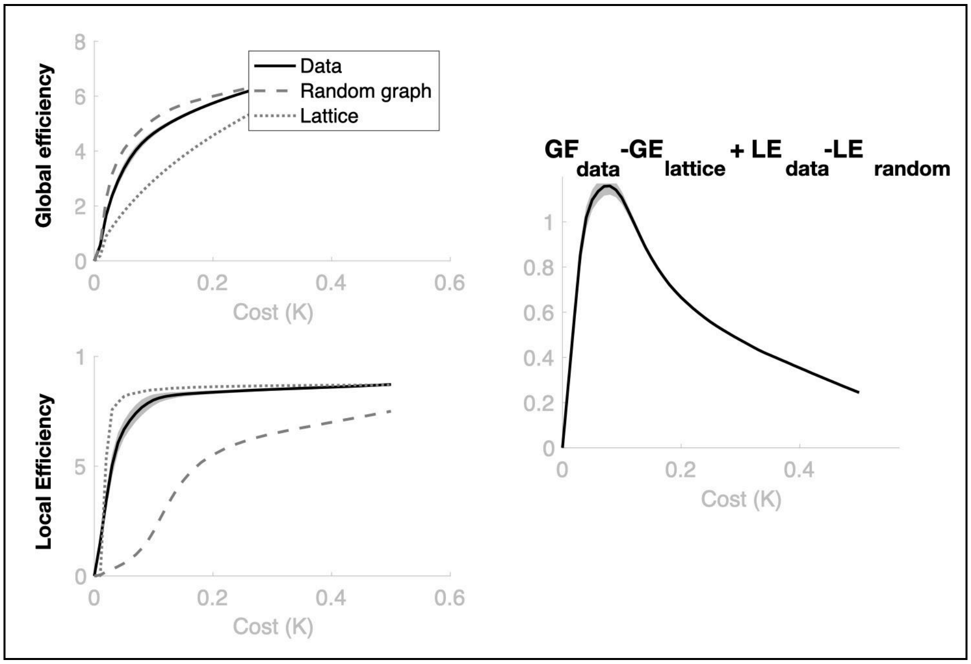

Supplementary Figure 5. Global and local efficiency of the structural network as a function of the cost threshold (k). The network exhibits small-world properties when it shows high global efficiency compared to a lattice graph and high local efficiency compared to a random graph (N=233).

Supplementary Tables

Supplementary Table 1. Descriptive statistics for MET and Gold-MSI subscales (N=232; 130 females).

	Mean	Std	Range
**MET**			Score Range
Total	73.51	8.86	53-94
Melody	35.85	5.49	24-49
Rhythm	37.77	4.60	25-48
**Gold-MSI**			Score Range (theoretical max)
Active Engagement	29.70	8.42	9-61 (63)
Perceptual abilities	41.44	7.33	10-59 (63)
Musical Training	13.34	8.28	2-42 (49)
Emotions	28.68	6.35	10-43 (49)
Singing Abilities	23.5	6.57	9-42 (42)
General Sophistication	58.19	14.46	28-107 (126)

Supplementary Table 2. Descriptive statistics for MET and Gold-MSI subscales (N=225; 125 females).

	Mean	Std	Range
**MET**			Score Range
Total	73.44	8.82	53-94
Melody	35.81	5.51	24-49

Rhythm	37.73	4.59	25-48
**Gold-MSI**			Score Range (theoretical max)
Active Engagement	29.76	8.45	9-61 (63)
Perceptual abilities	41.39	7.38	17-59 (63)
Musical Training	13.34	8.29	2-42 (49)
Emotions	28.64	6.36	10-43 (49)
Singing Abilities	23.53	6.56	9-42 (42)
General Sophistication	58.25	14.48	28-107 (126)

Supplementary Table 3. Descriptive statistics for MET, Gold-MSI subscales, and WAIS-IV tests (N=201; 150 females). This subset of participants was included in the regression analyses between functional connectomes and WAIS-IV scores.

	Mean	Std	Range
**MET**			Score Range
Total	73.27	8.58	53-91
Melody	35.61	5.33	24-49
Rhythm	37.64	4.54	25-48
**Gold-MSI**			Score Range (theoretical max)
Active Engagement	29.76	8.72	9-61 (63)
Perceptual abilities	41.69	7.19	21-58 (63)
Musical Training	13.57	8.31	2-37 (49)

Emotions	28.72	6.54	10-43 (49)
Singing Abilities	23.56	6.68	9-42 (42)
General Sophistication	58.06	14.95	28-17 (126)
**WAIS-IV**			
Verbal comprehension	109.87	10.51	83-137
Perceptual reasoning	113.99	13.37	78-150
Working memory	105.89	13.09	76-150
Processing speed	113.98	12.92	84-150
General Ability	112.37	10.01	83-142

Supplementary Table 4. Descriptive statistics for MET, Gold-MSI subscales, and WAIS-IV tests (N=195; 101 females). This subset of participants was included in the regression analyses between structural connectomes and WAIS-IV scores.

	Mean	Std	Range
**MET**			Score Range
Total	73.17	8.50	53-91
Melody	35.55	5.32	24-49
Rhythm	37.60	4.51	25.48
**Gold-MSI**			Score Range (theoretical max)
Active Engagement	29.80	8.75	9-61 (63)
Perceptual abilities	41.64	7.32	21-58 (63)

Musical Training	13.58	8.31	2-37 (49)
Emotions	28.63	6.48	10-43 (49)
Singing Abilities	23.58	6.66	9-42 (42)
General Sophistication	58.09	14.97	28-107 (126)
**WAIS-IV**			
Verbal comprehension	109.83	10.47	83-137
Perceptual reasoning	114.04	13.33	78-150
Working memory	105.90	13.10	76-150
Processing speed	114.10	13.00	84-150
General Ability	112.41	10.03	83-142

Supplementary Table 5. Table displays F-values from multiple regression analysis linking frontoparietal memory network graph metrics (structural connectivity) to percentage of MET total scores, adjusted for age, gender, and Musical Training Index (N=225). Asterisks denote significant relationships using a false discovery rate of q<0.05 (two-tailed), for each graph metric.

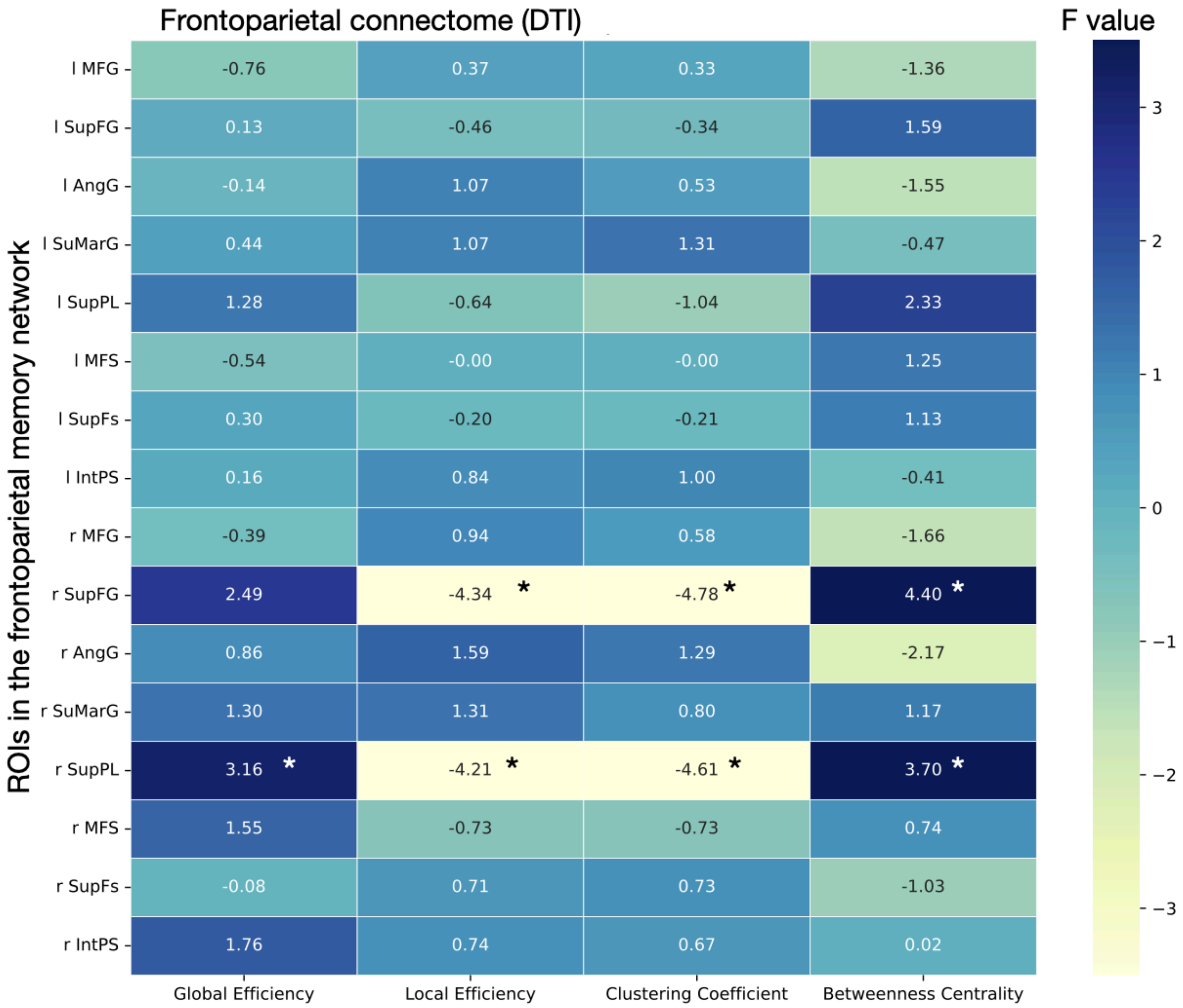

Supplementary Table 6. Table displays F-values from multiple regression analysis linking occipital control network graph metrics (structural connectivity) to percentage of MET total scores, adjusted for age, gender, and Musical Training Index (N=225). Asterisks denote significant relationships using a false discovery rate of q<0.05 (two-tailed), for each graph metric.

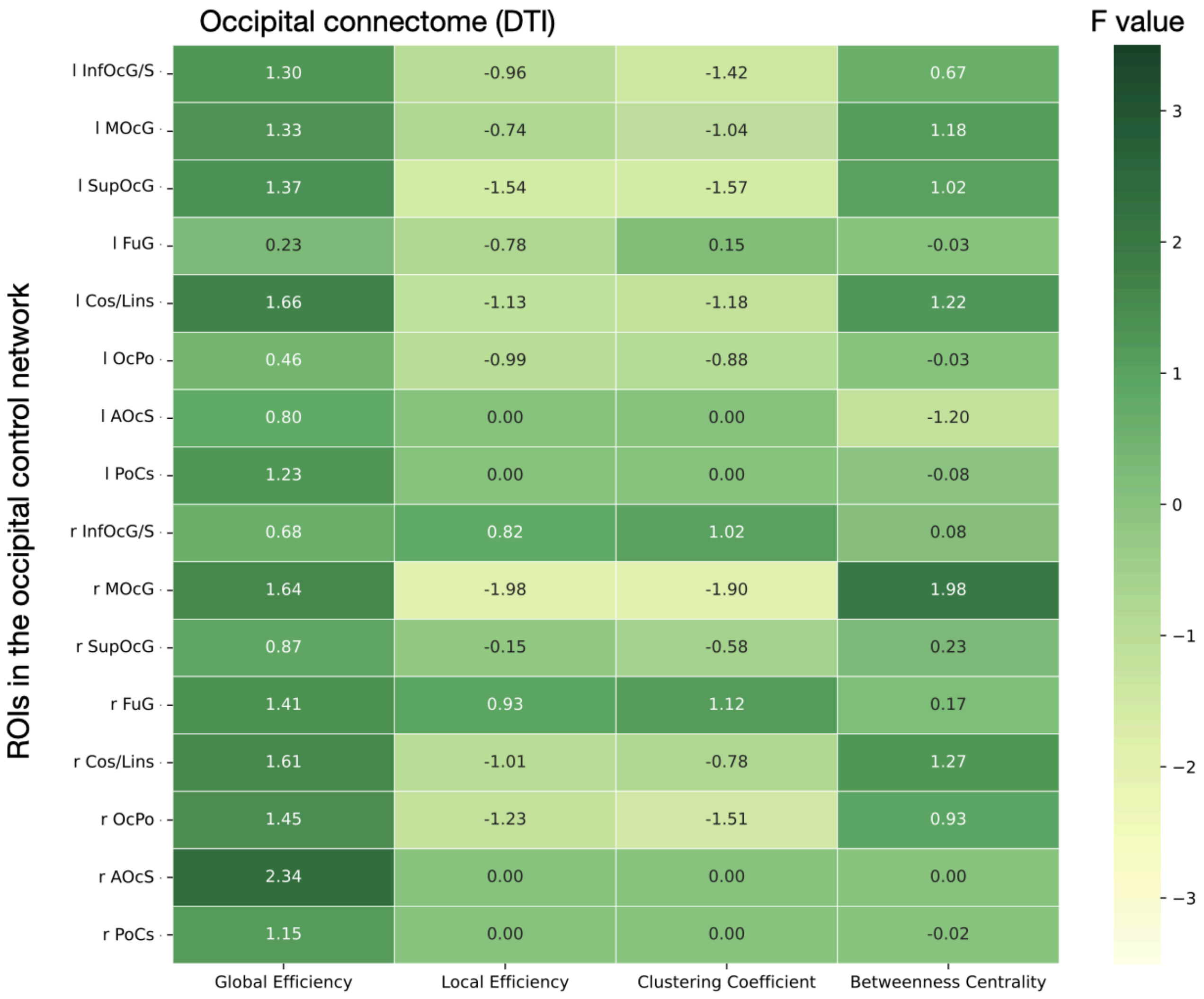

Supplementary Table 7. Table displays F-values from multiple regression analysis linking frontoparietal memory network graph metrics (functional connectivity) to percentage of MET total scores, adjusted for age, gender, and Musical Training Index (N=232). Asterisks denote significant relationships using a false discovery rate of q<0.05 (two-tailed), for each graph metric.

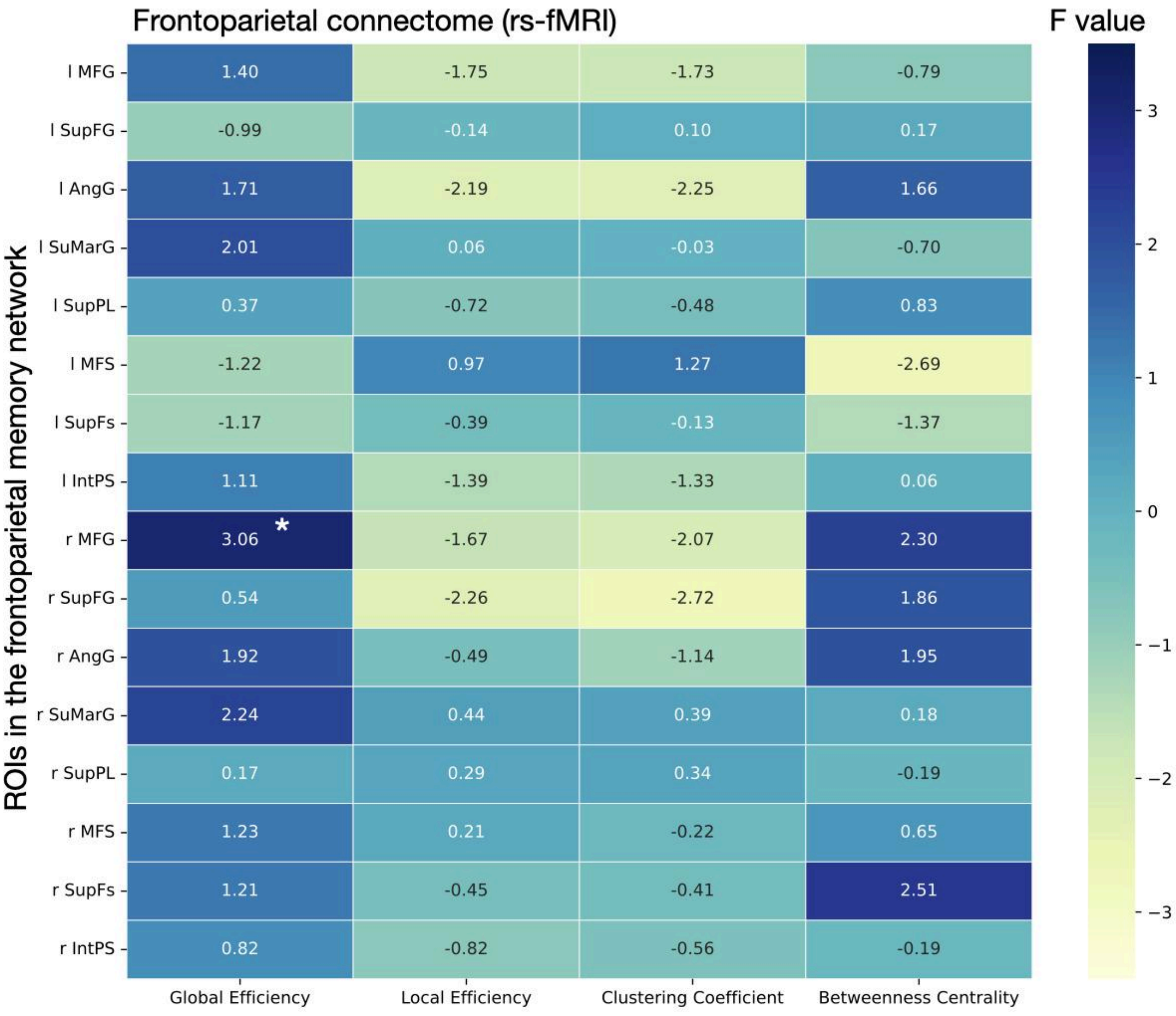

Supplementary Table 8. Table displays F-values from multiple regression analysis linking occipital control network graph metrics (functional connectivity) to percentage of MET total scores, adjusted for age, gender, and Musical Training Index (N=232). Asterisks denote significant relationships using a false discovery rate of q<0.05 (two-tailed), for each graph metric.

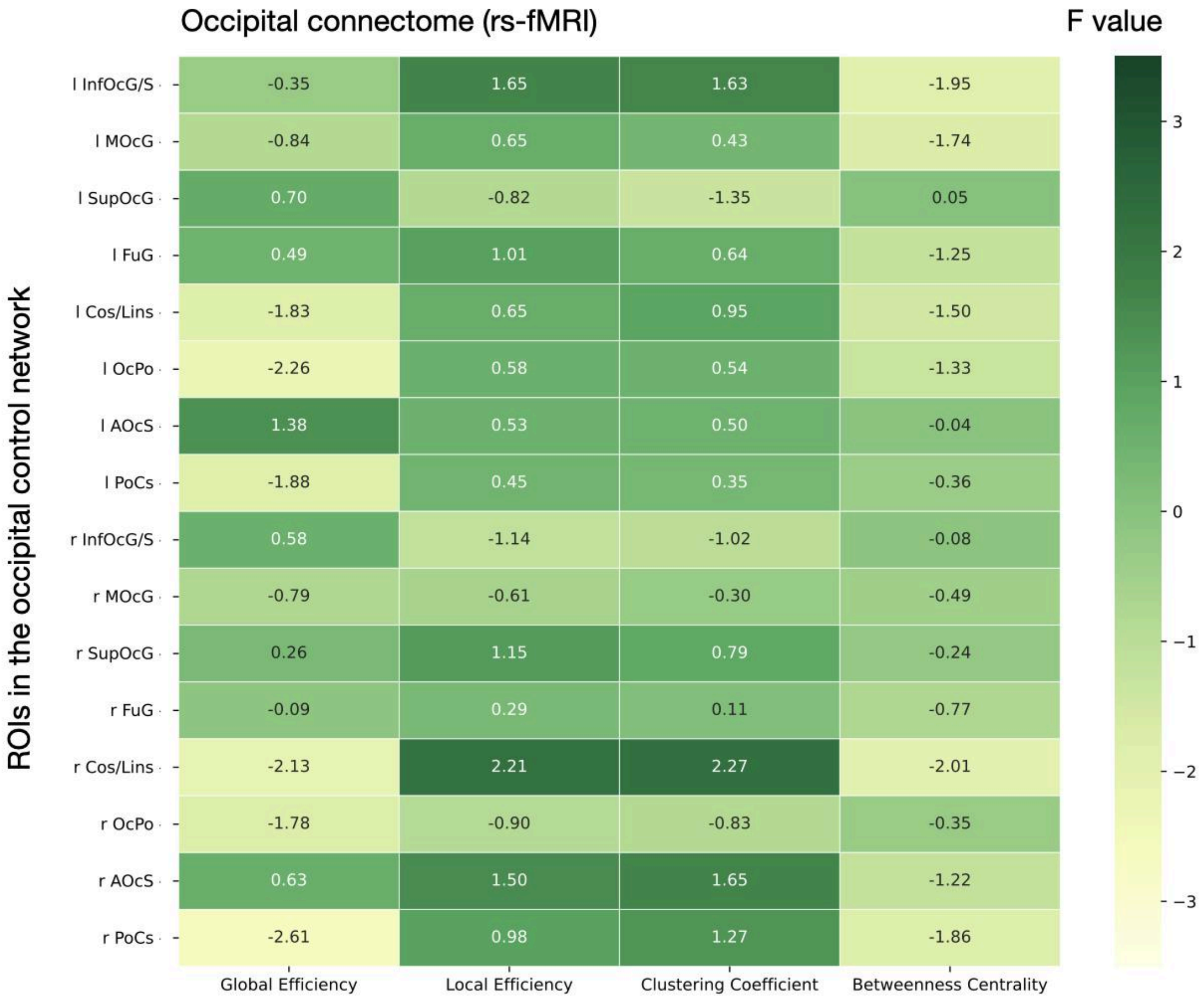

Supplementary Table 9. Table displays F-values from multiple regression analysis linking frontoparietal memory network graph metrics (functional connectivity) to WMI scores, adjusted for age, gender, and Musical Training Index (N=201). Asterisks denote significant relationships using a false discovery rate of q<0.05 (two-tailed), for each graph metric.

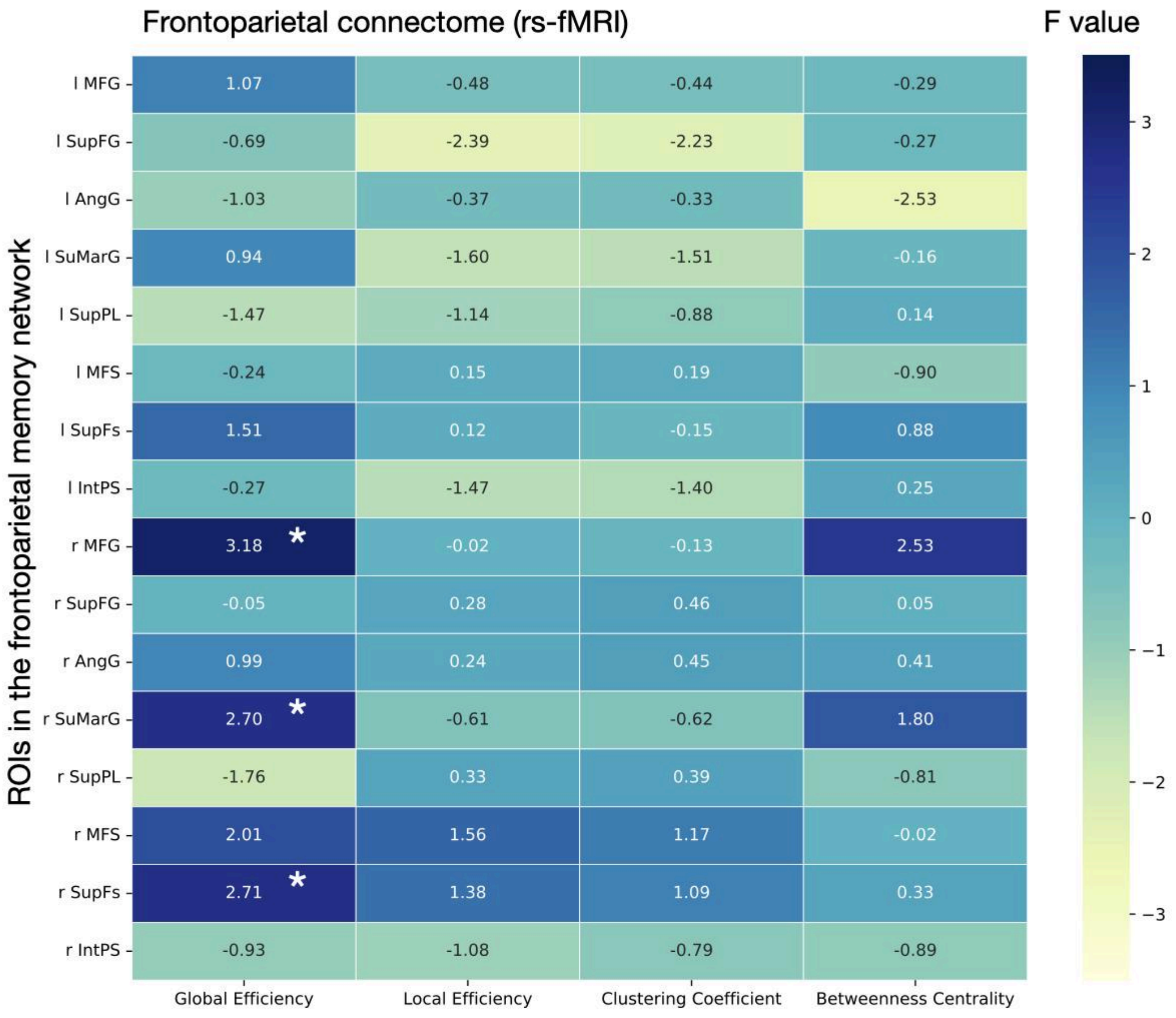

Supplementary Table 10. Table displays F-values from multiple regression analysis linking occipital control network graph metrics (functional connectivity) to WMI scores, adjusted for age, gender, and Musical Training Index (N=201). Asterisks denote significant relationships using a false discovery rate of q<0.05 (two-tailed), for each graph metric.

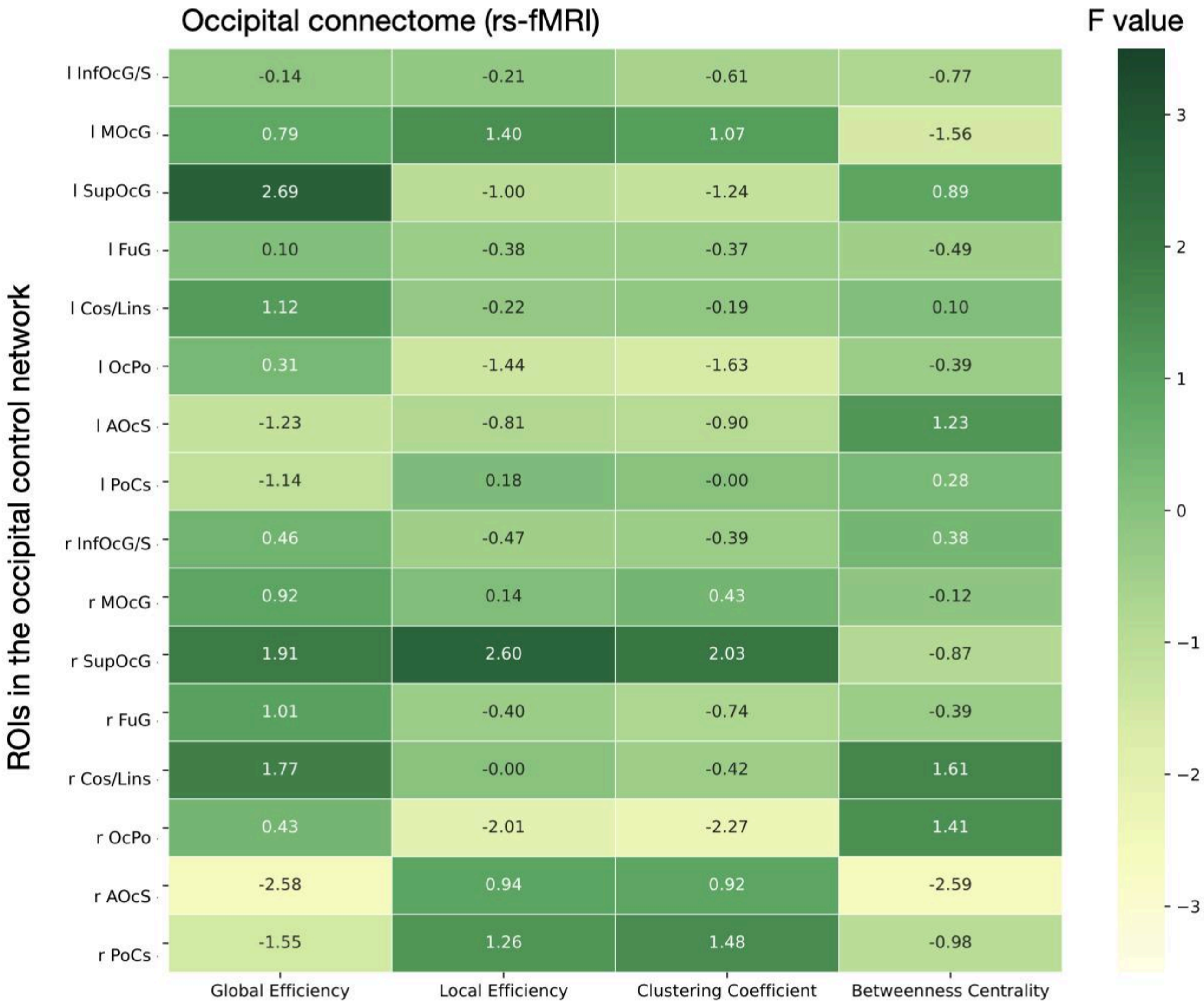

Supplementary Table 11. Table displays F-values from multiple regression analysis linking frontoparietal memory network graph metrics (structural connectivity) to WMI scores, adjusted for age, gender, and Musical Training Index (N=195). Asterisks denote significant relationships using a false discovery rate of q<0.05 (two-tailed), for each graph metric.

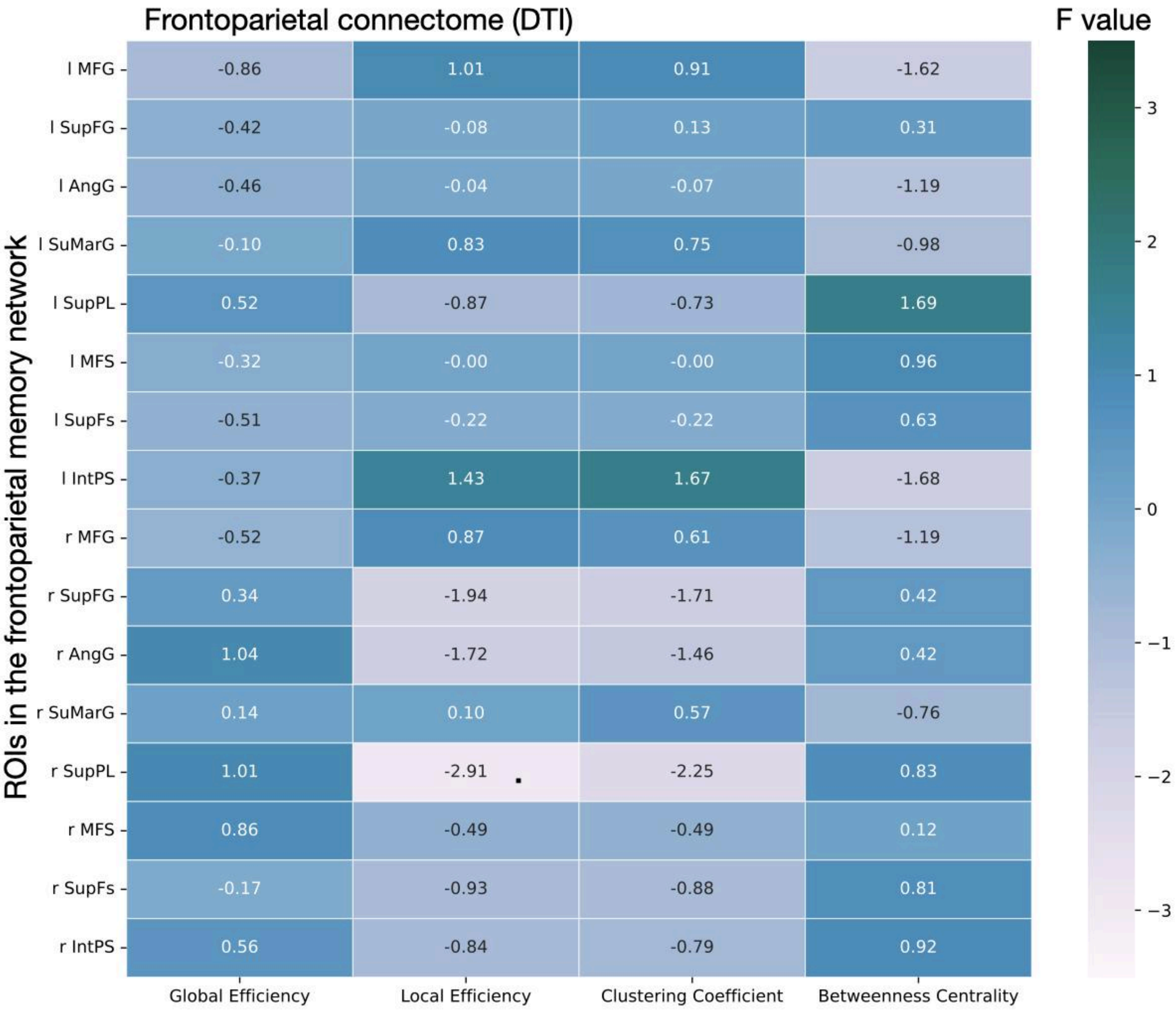

Supplementary Table 12. Table displays F-values from multiple regression analysis linking occipital control network graph metrics (structural connectivity) to WMI scores, adjusted for age, gender, and Musical Training Index (N=195). Asterisks denote significant relationships using a false discovery rate of q<0.05 (two-tailed), for each graph metric.

Occipital connectome (DTI) — F value

ROIs in the occipital control network	Global Efficiency	Local Efficiency	Clustering Coefficient	Betweenness Centrality
l InfOcG/S	-0.53	1.19	1.34	-1.66
l MOcG	0.34	-0.70	-0.85	0.66
l SupOcG	0.65	-0.39	-0.31	0.21
l FuG	0.42	-1.26	-0.57	-0.11
Cos/Lins	1.06	0.08	-0.24	1.68
l OcPo	0.96	-1.45	-1.72	0.60
l AOcS	0.45	0.00	0.00	0.37
l PoCs	0.47	0.00	0.00	0.59
InfOcG/S	0.58	0.95	1.06	-0.47
r MOcG	0.45	-0.20	0.58	-0.11
SupOcG	0.88	-0.45	0.11	0.81
r FuG	1.82	-0.45	-0.53	1.72
r Cos/Lins	0.14	0.49	1.09	1.05
r OcPo	0.60	-0.20	0.20	0.11
r AOcS	2.17	0.00	0.00	0.00
r PoCs	0.08	0.00	0.00	0.04

F value scale: 3, 2, 1, 0, −1, −2, −3

Supplementary Table 13. Table displays F-values from multiple regression analysis linking frontoparietal memory network graph metrics (functional connectivity) to Emotion (F5) scores, adjusted for age and gender (N=232). Asterisks denote significant relationships using a false discovery rate of q<0.05 (two-tailed), for each graph metric.

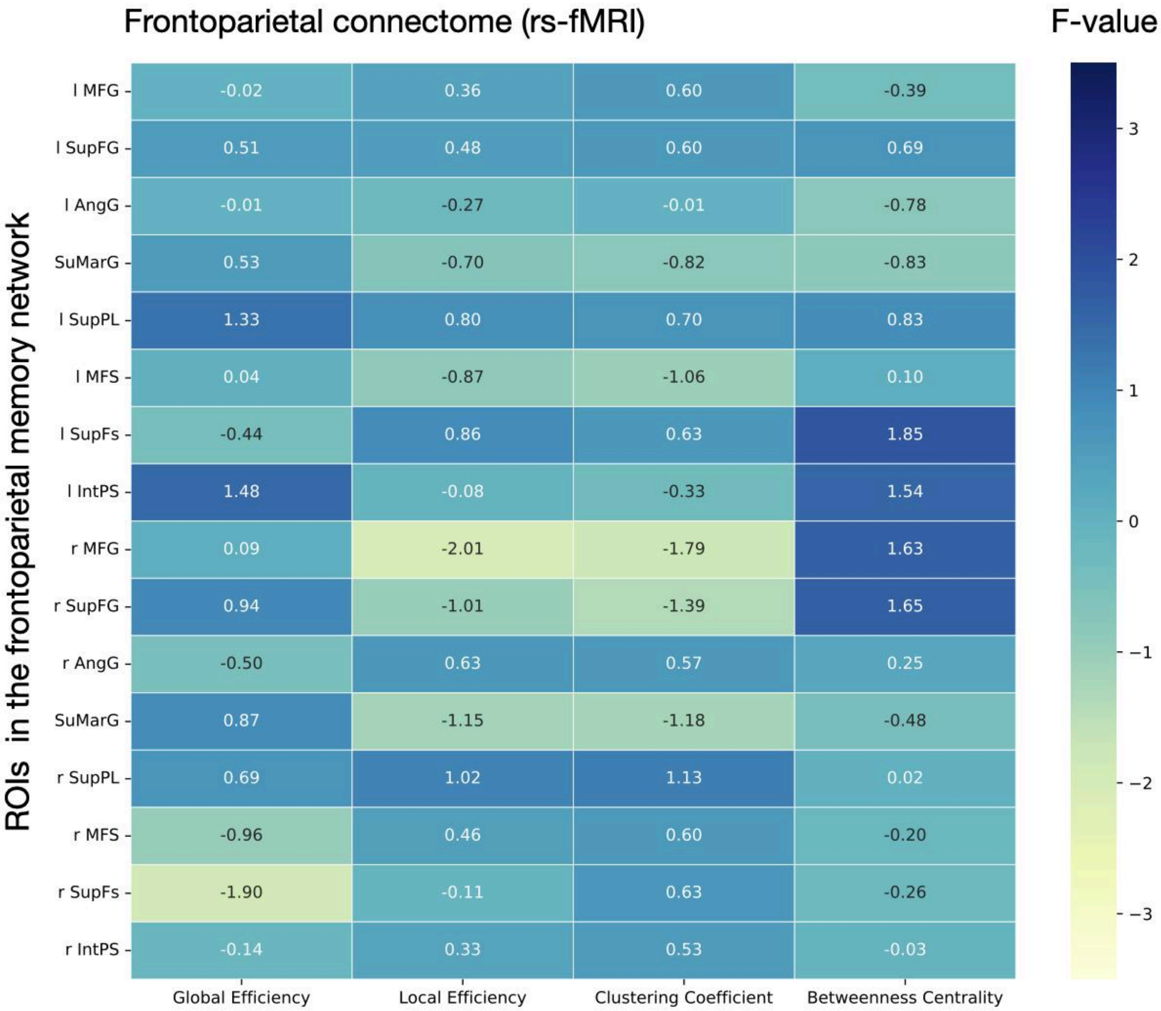

Supplementary Table 14. Table displays F-values from multiple regression analysis linking occipital network graph metrics (functional connectivity) to Emotion (F5) scores, adjusted for age and gender (N=232). Asterisks denote significant relationships using a false discovery rate of q<0.05 (two-tailed), for each graph metric.

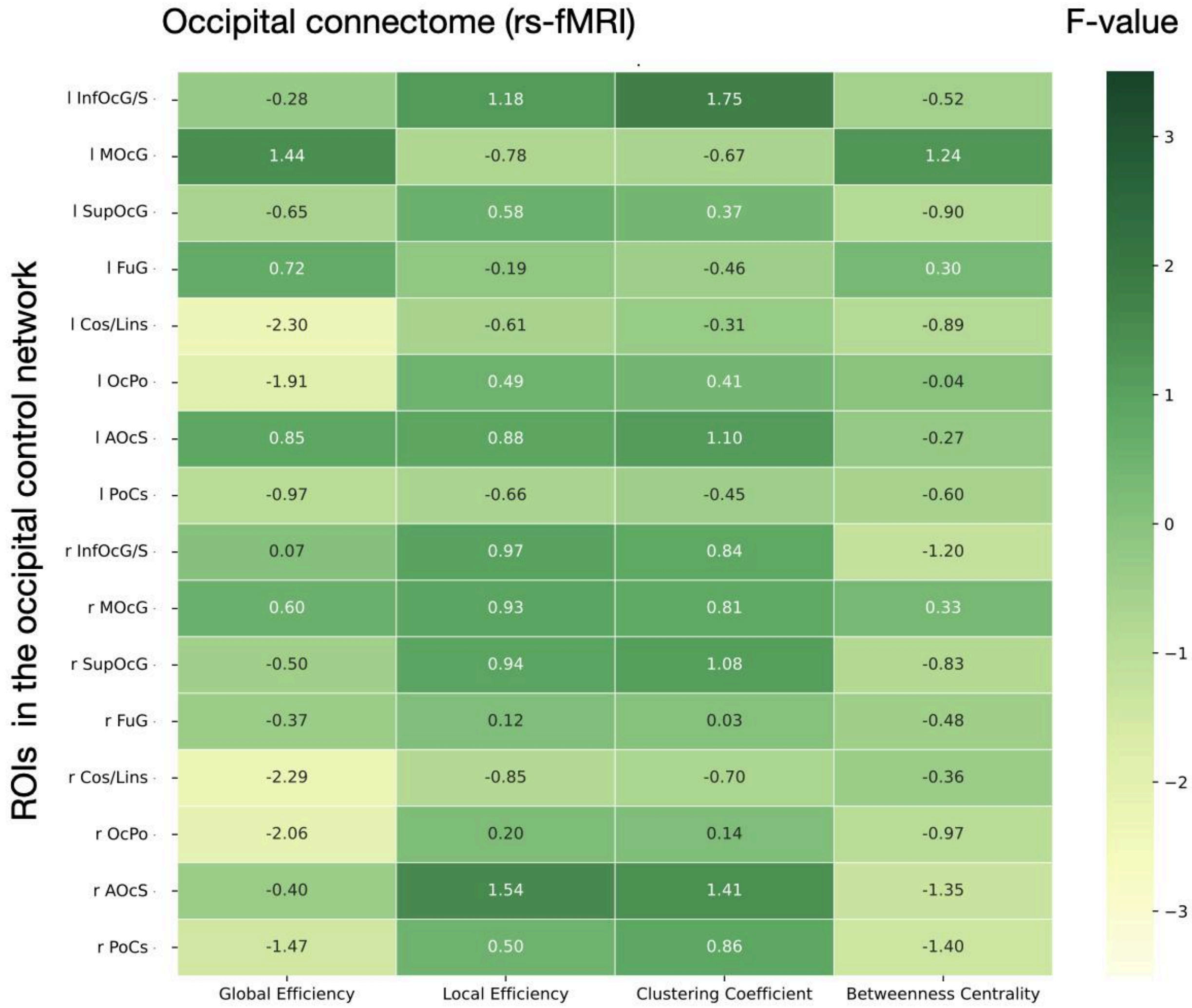

Supplementary Table 15. Table displays F-values from multiple regression analysis linking frontoparietal memory network graph metrics (structural connectivity) to Emotion (F5) scores, adjusted for age and gender (N=225). Asterisks denote significant relationships using a false discovery rate of q<0.05 (two-tailed), for each graph metric.

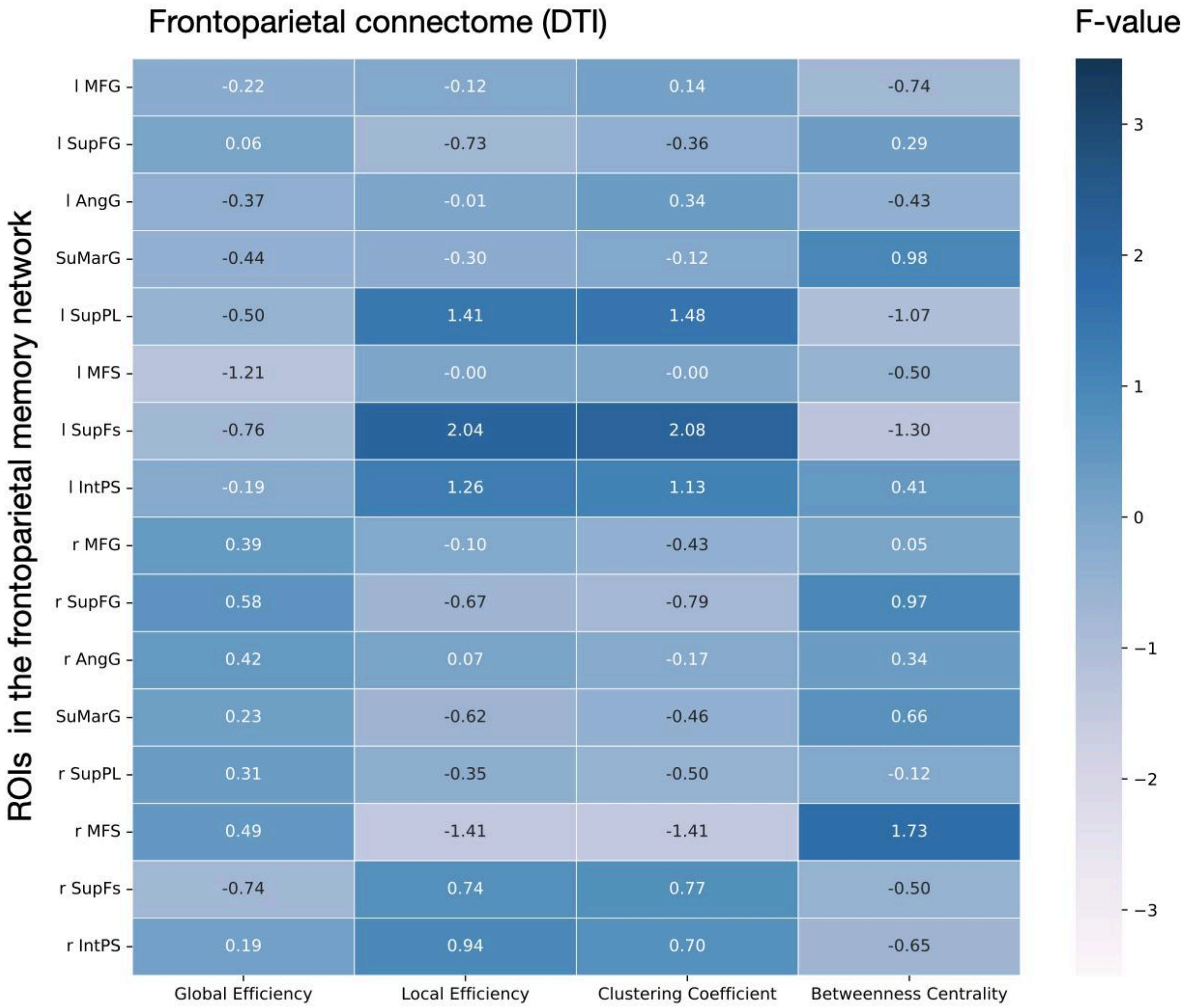

Supplementary Table 16. Table displays F-values from multiple regression analysis linking occipital network graph metrics (structural connectivity) to Emotion (F5) scores, adjusted for age and gender (N=225). Asterisks denote significant relationships using a false discovery rate of q<0.05 (two-tailed), for each graph metric.

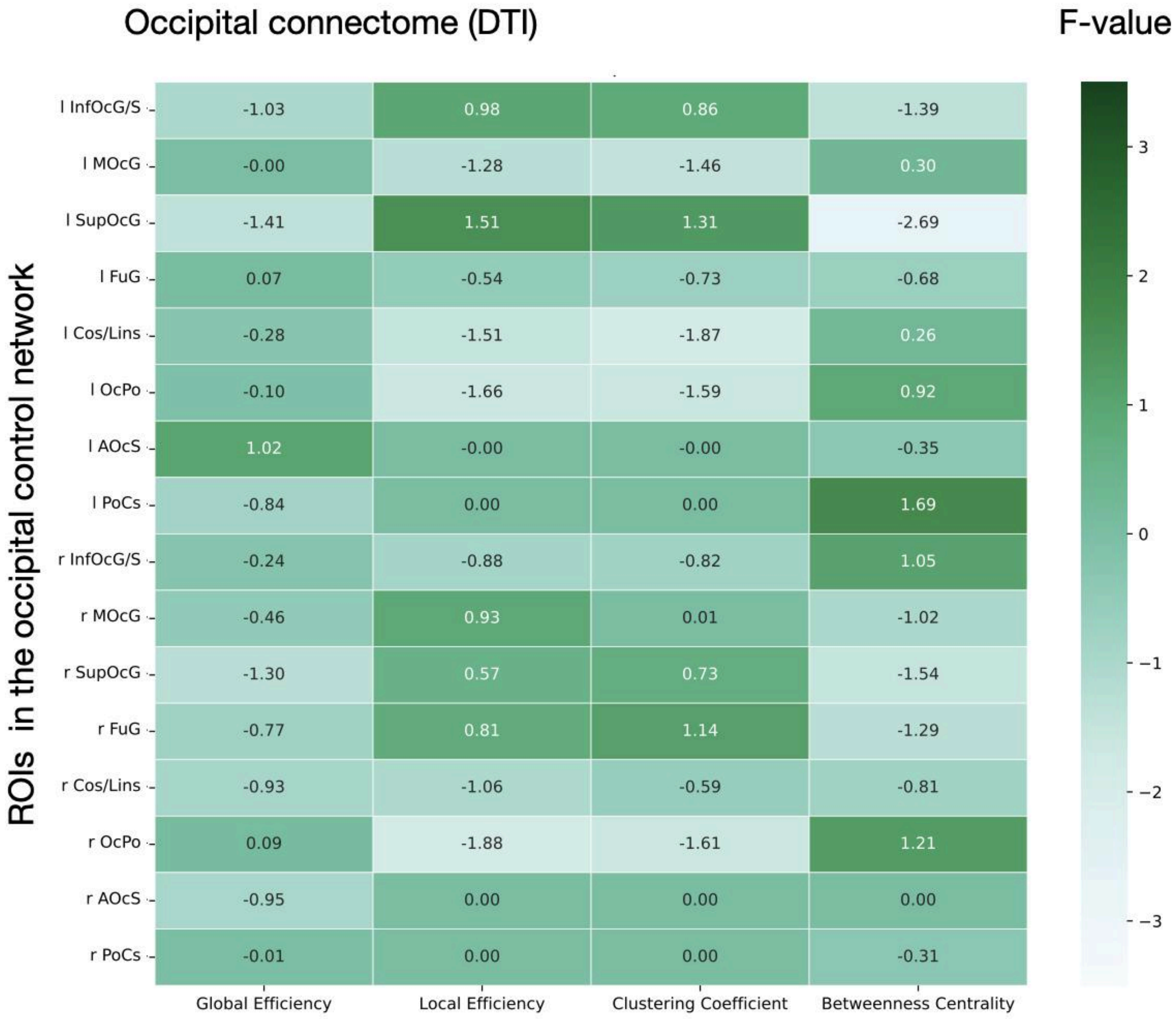

Supplement: Supplementary file 1 — Supplementary Material [file 41467_2024_52479_MOESM1_ESM.pdf]
