# Supplementary material for: Frontoparietal network topology as a neural marker of musical perceptual abilities
Source: Nat Commun. 2024 Sep 17;15:8160. doi: 10.1038/s41467-024-52479-z (PMC11408523; doi:10.1038/s41467-024-52479-z)
Supplement: Supplementary file 3 — Reporting Summary [file 41467_2024_52479_MOESM3_ESM.pdf]

nature portfolio

Corresponding author(s): Massimo Lumaca

Last updated by author(s): Aug 16, 2024

Reporting Summary

Nature Portfolio wishes to improve the reproducibility of the work that we publish. This form provides structure for consistency and transparency in reporting. For further information on Nature Portfolio policies, see our Editorial Policies and the Editorial Policy Checklist.

Statistics

For all statistical analyses, confirm that the following items are present in the figure legend, table legend, main text, or Methods section.

n/a	Confirmed	
☐	☒	The exact sample size (*n*) for each experimental group/condition, given as a discrete number and unit of measurement
☐	☒	A statement on whether measurements were taken from distinct samples or whether the same sample was measured repeatedly
☐	☒	The statistical test(s) used AND whether they are one- or two-sided<br>*Only common tests should be described solely by name; describe more complex techniques in the Methods section.*
☐	☒	A description of all covariates tested
☐	☒	A description of any assumptions or corrections, such as tests of normality and adjustment for multiple comparisons
☐	☒	A full description of the statistical parameters including central tendency (e.g. means) or other basic estimates (e.g. regression coefficient) AND variation (e.g. standard deviation) or associated estimates of uncertainty (e.g. confidence intervals)
☐	☒	For null hypothesis testing, the test statistic (e.g. *F*, *t*, *r*) with confidence intervals, effect sizes, degrees of freedom and *P* value noted<br>*Give P values as exact values whenever suitable.*
☒	☐	For Bayesian analysis, information on the choice of priors and Markov chain Monte Carlo settings
☒	☐	For hierarchical and complex designs, identification of the appropriate level for tests and full reporting of outcomes
☒	☐	Estimates of effect sizes (e.g. Cohen's *d*, Pearson's *r*), indicating how they were calculated

Our web collection on statistics for biologists contains articles on many of the points above.

Software and code

Policy information about availability of computer code

Data collection	All relevant behavioural data was collected with pen and paper.<br><br>The Musical Ear Test (MET) is a publicly accessible auditory assessment, provided in the form of an .mp3 file. This format ensures broad compatibility, allowing the test to be easily administered using any standard media player software. Designed to evaluate musical perception and aptitude, the MET offers an accessible approach to measuring musical abilities.<br><br>The Gold-MSI is a self-report inventory assessing individual differences in musical sophistication. It measures the ability to engage with music in a flexible, effective and comprehensive way. This assessment can be easily conducted using just paper and pen, making it accessible for a wide range of participants.<br><br>WAIS-IV is the most advanced adult measure of cognitive ability.<br>It consists of 10 core subtests (Block Design, Matrix Reasoning, Visual Puzzles, Digit Span, Arithmetic, Similarities, Vocabulary, Information, Symbol Search, and Coding) that yield scores on four factors, as well as a full IQ score.
Data analysis	Neuroanatomical data analysis: fMRIPrep 21.0.2 (RRID:SCR_016216), Nipype 1.6.1 (RRID:SCR_002502), ANTs 2.3.3(RRID:SCR_004757), FSL 6.0.5.1 (RRID:SCR_002823), Freesurfer 6.0.1 (RRID:SCR_001847), Mindboggle (RRID:SCR_002438)<br><br>Diffusion Analysis: Mrtrix3, FSL 6.0.5.1<br><br>Resting-state fMRI analysis: CONN (RRID:SCR_009550) release 21.a, SPM (RRID:SCR_007037) release 12.7771, Matlab 2016b

Graph Theory Analysis: Matlab 2016.b, Brain Connectivity Toolbox (BCT, Version 2019-03-03), CONN (RRID:SCR_009550) release 21.a

Code for reproducing graph theory results is available on GitHub: https://github.com/MassimoLumaca/neuroMET. Code for reproducing diffusion analysis is available on GitHub: https://github.com/MassimoLumaca/neuroARC.

For manuscripts utilizing custom algorithms or software that are central to the research but not yet described in published literature, software must be made available to editors and reviewers. We strongly encourage code deposition in a community repository (e.g. GitHub). See the Nature Portfolio guidelines for submitting code & software for further information.

Data

Policy information about availability of data

All manuscripts must include a data availability statement. This statement should provide the following information, where applicable:

- Accession codes, unique identifiers, or web links for publicly available datasets
- A description of any restrictions on data availability
- For clinical datasets or third party data, please ensure that the statement adheres to our policy

Provide your data availability statement here.

Research involving human participants, their data, or biological material

Policy information about studies with human participants or human data. See also policy information about sex, gender (identity/presentation), and sexual orientation and race, ethnicity and racism.

Reporting on sex and gender	Biological sex was self-reported by the participants. In the GLM regression analysis employed for examining the relationship between graph theory metrics and behavioural scores, biological sex was incorporated as a covariate. It is pertinent to note that data regarding participants' gender identity were not solicited, given its lack of relevance to the objectives of the present study. The composition of our primary sample includes 135 individuals identifying as female and 105 as male.
Reporting on race, ethnicity, or other socially relevant groupings	Information pertaining to race, ethnicity, or other socially relevant demographics was not solicited, given their lack of relevance to the objectives of the present study.
Population characteristics	Age and biological sex
Recruitment	Recruitment of participants was conducted via the Center of Functionally Integrative Neuroscience (CFIN) at Aarhus University, leveraging both the university's participant database and local advertising. No self-selection bias is reported: All volunteers who did not meet any of the exclusion criteria were included in the study. All volunteers signed an informed consent.
Ethics oversight	The project protocol received ethical approval from De Videnskabsetiske Komitéer for Region Midtjylland, Denmark.

Note that full information on the approval of the study protocol must also be provided in the manuscript.

Field-specific reporting

Please select the one below that is the best fit for your research. If you are not sure, read the appropriate sections before making your selection.

☒ Life sciences ☐ Behavioural & social sciences ☐ Ecological, evolutionary & environmental sciences

For a reference copy of the document with all sections, see nature.com/documents/nr-reporting-summary-flat.pdf

Life sciences study design

All studies must disclose on these points even when the disclosure is negative.

Sample size	Detailed sample size considerations for sufficient statistical power are elaborated in Section 1.3.2 of the Technical Annex of the Action, which can be accessed here: https://e-services.cost.eu/files/domain_files/CA/Action_CA18106/mou/CA18106-e.pdf. Figure 1a shows that to make a single comparison, more than 40 participants are needed. To correct for brain-wide multiple comparisons, alpha is typically not adjusted according to the number of actual comparisons (e.g. voxels), but rather according to the expected rate of false positives. Even with this in mind, however, N would need to be at least 200 in many cases to conduct a sufficiently powered study. In the context of the present study, where we target a network-of-interest, an even smaller sample size is in principle sufficient to achieve robust statistical power.
Data exclusions	All participants with personal history of neurological or psychiatric disorders and with hearing deficits were excluded from the study.
Replication	No replication test was performed given the large sample size used in this study (N>200).
Randomization	The allocation of participants was not randomized. Participants from Aarhus University and the local community were recruited, ensuring a diverse population in terms of age, biological sex, and musical sophistication, with no neurological or psychiatric disorders and no hearing deficits.

	To control for covariates, we included age, sex, and musical training as nuisance regressors in our second-level General Linear Model (GLM) analysis. This approach allowed us to account for potential confounding variables that could influence the relationship between the frontoparietal network topology and musical perceptual abilities.
Blinding	The study did not involve group allocation.

Reporting for specific materials, systems and methods

We require information from authors about some types of materials, experimental systems and methods used in many studies. Here, indicate whether each material, system or method listed is relevant to your study. If you are not sure if a list item applies to your research, read the appropriate section before selecting a response.

Materials & experimental systems

n/a	Involved in the study
☒	☐ Antibodies
☒	☐ Eukaryotic cell lines
☒	☐ Palaeontology and archaeology
☒	☐ Animals and other organisms
☒	☐ Clinical data
☒	☐ Dual use research of concern
☒	☐ Plants

Methods

n/a	Involved in the study
☒	☐ ChIP-seq
☒	☐ Flow cytometry
☐	☒ MRI-based neuroimaging

Plants

Seed stocks	n/a
Novel plant genotypes	n/a
Authentication	n/a

Magnetic resonance imaging

Experimental design

Design type	Resting state design
Design specifications	Each participant underwent to the following scanning design. Following an initial scout scan, two resting-state fMRI sequences (12 and 6 minutes) were run, accompanied by quantitative multi-parameter mapping (around 20 minutes) — used here for synthetically generated T1-weighted images— and high-angular resolution diffusion imaging (HARDI) (around 10 minutes), within a one-hour scanning session
Behavioral performance measures	No behavioural responses were recorded during the scans

Acquisition

Imaging type(s)	resting-state functional MRI (rs-fMRI), multi-parametric maps (MPMs), and high-angular resolution diffusion imaging (HARDI)
Field strength	3 Tesla
Sequence & imaging parameters	Resting state fMRI: For each participant, 1500 functional volumes were acquired (TR, 700 ms; TE, 30 ms; voxel size 2.5 mm3).<br><br>Multi-parametric maps (MPMs): The acquisition protocol had the following parameters): TR of PD- and T1-weighted contrasts: 18 ms; TR of MT-weighted contrast: 37 ms; minimum/maximum TE of PD-, T1- and MT-weighted contrasts: 2.46/14.76 ms; flip angles for MT-, PD- and T1-weighted contrasts: 6°, 4°, 25°, respectively; six equidistant echoes; 1 mm isotropic reconstruction voxel size; Field of view 224´ 256´ 176 mm; AP phase encoding direction; GRAPPA parallel imaging speedup factor of 2; T1w, PDw and MTw acquisition times: 3:50, 3.50, 7.52.
Area of acquisition	Whole-brain

Diffusion MRI ☒ Used ☐ Not used

Parameters: High-angular Resolution Diffusion Images (HARDI), multishell: 75 diffusion directions at b = 2500 s/mm2; 60 directions at b = 1500 s/mm2; 21 directions at b = 1200 s/mm2; 30 directions at b = 1000 s/mm2; 15 directions at b = 700/mm2; 10 directions at b = 5 s/mm2, with the different b-shells acquired in the same series (flip angle = 90∘, TR/TE = 2850/71 ms, voxel size = 2 mm3; matrix size = 100 x 100, number of slices = 84). The phase-encoding direction was anterior to posterior (AP). An opposite phase-encoding direction (PA) was also acquired (b = 0 s/mm2) to allow EPI distortion correction.

Preprocessing

Preprocessing software

MPMs preprocessing: fMRIPrep 21.0.2 (RRID:SCR_016216), Nipype 1.6.1 (RRID:SCR_002502), ANTs 2.3.3(RRID:SCR_004757), FSL 6.0.5.1; RRID:SCR_002823, ANTs 2.3.3. Details of their use can be found in the manuscript.

HARD preprocessingI: The diffusion MRI (dMRI) data was preprocessed using custom MATLAB scripts developed internally at the Center of Functionally Integrative Neuroscience (CFIN). These scripts are available on request.

Resting-state fMRI preprocessing: CONN (RRID:SCR_009550) release 21.a, SPM (RRID:SCR_007037) release 12.7771. Functional data were realigned using SPM realign & unwarp procedure where all scans were coregistered to a reference image (first scan of the first session) using a least squares approach and a 6 parameter (rigid body) transformation, and resampled using b-spline interpolation to correct for motion and magnetic susceptibility interactions. Potential outlier scans were identified using ART (see below), and a reference BOLD image was computed for each subject by averaging all scans excluding outliers. Functional and anatomical data were coregistered using SPM intermodality coregistration procedure with a normalised mutual information objective function. After surface-based normalization (see below), functional data were smoothed using 40 iterative diffusion steps, approximately a 8 mm FWHM smoothing kernel within the cortical surface.

Normalization

Anatomical: Volume-based spatial normalization of the brain images to the two standard spaces (MNI152NLin2009cAsym, MNI152NLin6Asym) was executed through nonlinear registration with antsRegistration (ANTs 2.3.3), using brain-extracted versions of the T1w reference and the T1w template.

HARDI: data were not normalized

rs-fMRI: surface-based normalization. Functional images were resampled at the cortical surface by averaging the functional data from ten locations between the pial and white matter cortical surfaces of each subject at each individual vertex in FreeSurfer fsaverage level-8 tessellation, with 163,842 vertices per hemisphere.

Normalization template

Anatomical: ICBM 152 Nonlinear Asymmetrical template version 2009c; FSL's MNI ICBM 152 non-linear 6th Generation Asymmetric Average Brain Stereotaxic Registration Model146

rs-fMRI Fsaverage (Freesurfer's standard template)

Noise and artifact removal

Anatomical: The synthetic T1-weighted image was preprocessed using fMRIPrep 21.0.2139 (RRID:SCR_016216), which is based on Nipype 1.6.1140 (RRID:SCR_002502). The T1-weighted image were corrected for intensity non-uniformity (INU) using the N4BiasFieldCorrection141, part of the ANTs 2.3.3142 (RRID:SCR_004757).

rs-fMRI: functional data were denoised using a standard denoising pipeline. including the regression of potential confounding effects characterized by white matter timeseries (5 CompCor noise components), CSF timeseries (5 CompCor noise components), motion parameters and their first order derivatives (12 factors), outlier scans (below 148 factors) session effects and their first order derivatives (2 factors), and linear trends (2 factors) within each functional run, followed by bandpass frequency filtering of the BOLD timeseries between 0.008 Hz and 0.09 Hz.

HARDI: The preprocessing of HARDI images included noise reduction, correction of Gibbs ringing artifacts, and motion, eddy currents, and field distortion corrections using the top-up and eddy tools from the FSL toolbox

Volume censoring

rs-fMRI: Potential outlier scans were identified using ART as acquisitions with framewise displacement above 0.9 mm or global BOLD signal changes above 5 standard deviations.

Statistical modeling & inference

Model type and settings

rs-fMRI (first-level). ROI-to-ROI connectivity (RRC) matrices were estimated characterizing the functional connectivity between each pair of regions among 148 cortical ROIs of the Destrieux parcellation. Functional connectivity strength was represented by Fisher-transformed bivariate correlation coefficients from a general linear model (weighted-GLM), estimated separately for each pair of ROIs, characterizing the association between their BOLD signal timeseries. In order to compensate for possible transient magnetization effects at the beginning of each run, individual scans were weighted by a step function convolved with an SPM canonical hemodynamic response function and rectified.

DWI(first-level): 5-tissue-type (5tt) images were created from preprocessed DWI data containing masks of different tissue types within the brain ((cortical grey matter, deep grey matter, white matter, CSF and "other"). Co-registration was then performed to align T1-weighted and DWI images. A response function was created for each major tissue type (white matter, grey matter, cerebrospinal fluid) for each participant. The individual participant response functions were used to create group-level response functions. Multi-Shell Multi-Tissue Constrained Spherical Deconvolution (MSMT-CSD), was used to estimate Fiber Orientation Distributions (FODs) within each voxel of the brain, followed by normalisation. Next, whole-brain probabilistic tractography was performed using the ACT framework and backtracking. The maximum attempted number of

streamlines was 1*10^ qi9 streamlines with 10 million streamlines per brain network being selected. Each seed was determined dynamically from the FOD image using the SIFT model. The FOD cutoff was 0.06, the maximum length of each selected streamlines was 250mm while the minimum was 20mm. The SIFT2 model was then applied to the data. Structural networks for each subject were then generated using the Destrieux parcellation for the cortex and the FSL FIRST segmentations for the subcortical structures.

Second-level analysis (functional and structural data): multiple regression with GLM (mass univariate: analysis performed for each node of the network of interest). Fix effects were MET scores or WMI scores. Covariates were age, biological sex, and the musical training indexes. The dependent variable were graph theory metrics (Global Efficiency, Local Efficiency, Clustering Coefficient, or Betweenness Centrality).

Effect(s) tested

Test how changes in the fixed effect affect the dependent variable after controlling for the influence of covariates

Specify type of analysis: ☐ Whole brain ☒ ROI-based ☐ Both

Anatomical location(s)

Anatomical locations were determined with automated neuroanatomical parcellation/labeling in Freesurfer, resulting in 148 cortical ROIs (Destrieux's atlas). Sixteen ROIs were then selected to define a frontoparietal network (main analysis) or a visual network (control analysis).

Statistic type for inference

(See Eklund et al. 2016)

Node-wise statistics

Correction

Node-level p-values were adjusted for multiple comparisons (N=16) using a false discovery rate of q<0.05 (two-sided), for each graph metric.

Models & analysis

n/a	Involved in the study
☐	☐ Functional and/or effective connectivity
☐	☒ Graph analysis
☐	☐ Multivariate modeling or predictive analysis

Functional and/or effective connectivity

n/a

Graph analysis

We used Fisher-transformed bivariate correlation coefficients for functional connectomes and Fiber Bundle Capacity (FBC) for structural connectomes. We constructed binarized and undirected graphs using proportional thresholding. Notably, our functional graphs included both positive and negative resting-state functional connectivity (rs-FC) values. Binary undirected matrices were calculated for each subject. Node-level metrics were calculated from these matrices, including clustering coefficient, local efficiency, global efficiency, and betweenness centrality.

Multivariate modeling and predictive analysis

n/a
